# What Are the Major Determinants in the Success of Smoking Cessation: Results from the Health Examinees Study

**DOI:** 10.1371/journal.pone.0143303

**Published:** 2015-12-03

**Authors:** Jae Jeong Yang, Minkyo Song, Hyung-Suk Yoon, Hwi-Won Lee, Yunhee Lee, Sang-Ah Lee, Ji-Yeob Choi, Jong-koo Lee, Daehee Kang

**Affiliations:** 1 Department of Preventive Medicine, Seoul National University College of Medicine, Seoul, Korea; 2 Institute of Environmental Medicine, Seoul National University Medical Research Center, Seoul, Korea; 3 Department of Biomedical Sciences, Seoul National University Graduate School, Seoul, Korea; 4 Department of Preventive Medicine, Kangwon National University, Kangwon-do, Korea; 5 Cancer Research Institute, Seoul National University, Seoul, Korea; 6 JW Lee Center for Global Medicine, Seoul National University College of Medicine, Seoul, Korea; 7 Department of Family Medicine, Seoul National University College of Medicine, Seoul, Korea; Duke Cancer Institute, UNITED STATES

## Abstract

Understanding mechanisms underlying smoking-related factors should be prioritized in establishing smoking prevention and cessation policy. The aim of this study was to identify factors significantly associated with smoking initiation and/or smoking cessation as well as the most important determinants of successful smoking cessation in a developed non-Western setting. Based on multiple logistic regression models, the odds ratios (ORs) for smoking initiation and cessation were estimated among males (*N* = 24,490) who had participated in the Health Examinees (HEXA) study. The Cox proportional hazards regression model was used to assess the association between selected predictors of smoking cessation and the likelihood of reaching this goal. Finally, Kaplan–Meier curves were constructed to illustrate the distribution of time from age at smoking initiation to age at smoking cessation. We found that the ORs for successfully quitting smoking increased with age, married status, educational achievement, having a non-manual job, drinking cessation and disease morbidity. Those exposed to secondhand smoking showed less likelihood of quitting smoking. A continual decrease in the ORs for successfully quitting smoking was observed according to increased smoking duration, smoking dose per day and lifetime tobacco exposure (*p*_trend_ <0.001). Among the selected predictors, lifetime tobacco exposure, educational attainment, alcohol drinking status and birth cohort were the major determinants in the success of smoking cessation. Our findings suggest that lifetime tobacco exposure, educational attainment, alcohol drinking status and birth cohort can determine success in smoking cessation. Public interventions promoting a smoke-free environment are needed to reinforce discouraging the initiation of, reducing, and quitting cigarette smoking.

## Introduction

Cigarette smoking, which is the second leading contributory factor to diseases worldwide, remains a potent threat to the public health [1]. Each year, nearly six million deaths and hundreds of billions of dollars in health expenditure are attributed to cigarette smoking globally [2]. It is estimated that if the current trend continues, smoking-related deaths will increase to more than 8 million per year by 2030 [2].

Despite the damage caused by cigarette smoking, the prevalence rate of smoking has not fallen dramatically worldwide. To control the smoking epidemic, many countries have taken concerted actions, such as anti-tobacco campaigns, a tax increase on cigarettes, and comprehensive smoking cessation interventions [3, 4]; yet, tobacco exposure remains a substantial problem. Furthermore, there are emerging concerns that the proportion of smokers continues to decline but at a slower rate in developed countries and that prevalence rates are increasing in lower- and middle-income countries. In particular, many Asian countries have faced diverse challenges regarding tobacco control and prevention: Asian men showed the highest global prevalence of smoking, and factors such as low smoking cessation rate, increasing female smoking prevalence, and aggressive marketing strategies by tobacco companies are other smoking-related challenges faced by the Asian population [5, 6]. This means that the latent damage caused by cigarette smoking will continue to be a threat to the public health worldwide and greater attention should be paid to the non-Western populations.

It is generally assumed that the hazards of smoking exposure can be diminished simply by quitting smoking, and long-term health benefits of cessation have been widely reported [7–9]. Therefore, smoking prevention and cessation should be a major target for health policy, and understanding mechanisms underlying smoking-related factors should be prioritized in establishing smoking prevention and cessation policy. To develop tailor-made interventions and to convert smokers to quitters, first of all, individual characteristics that predict success in smoking cessation need to be identified by considering cultural and social diversity. Sociocultural context may be the key to understanding barriers to tobacco control and prevention [10]: it is generally accepted that sociocultural contexts influence an individual’s perceived norm towards smoking, which in turn can affect smoking attitudes and behaviours. This can eventually induce different smoking-related cycles across the life course. However, most studies regarding determinants of smoking cessation have been conducted in the Western population [11–17]; thus it is necessary to identify factors representative of people in the non-Western population.

In the present study, using data derived from a developed non-Western population, we attempted to 1) identify characteristics significantly associated with smoking initiation and/or smoking cessation and 2) evaluate which of the putative predictors constituted the most important determinants in smoking cessation in a large-scale cross-sectional analysis.

## Materials and Methods

### Study population

This study is based on a large-scale genomic cohort, the Health Examinees (HEXA) Study, in Korea. The HEXA study was approved by the Ethics Committee of the Korean Health and Genomic Study of the Korean National Institute of Health. Detailed descriptions of the HEXA study are available in the literature [18]. Briefly, healthy volunteers were prospectively recruited and signed an informed consent form before enrolment. Following a standardised study protocol, all participants completed the questionnaire survey to collect information on individual characteristics, socio-demographic factors, past disease history, medication use, lifestyles, sexual maturation and dietary habits. Biological samples of plasma, serum, buffy coat, blood cells, genomic DNA and urine were collected. Physical examinations and laboratory analyses were also performed by skilful medical staff.

At the first phase of the HEXA study between 2004 and 2008, a total of 85,323 subjects aged 40–69 years were recruited. To define the analytic study population, 28,075 males were preliminarily screened, whereas females were excluded because almost all females were non-smokers (>98%). Of these, males were excluded if the following information was unavailable: smoking status (*n* = 285), age of smoking initiation (*n* = 670), smoking duration (*n* = 478), number of cigarettes smoked per day (*n* = 356) and specific information on smoking cessation (*n* = 1,796). Using the above criteria, 24,490 males who fulfilled all the inclusion criteria in the entire dataset were included in the study.

### Ascertainment of smoking history

Smoking status was ascertained by posing the following question: “Have you smoked more than 20 packs of cigarettes (400 cigarettes) in your lifetime?” Subjects who responded as never having smoked 400 cigarettes were defined as never smokers, quitters were defined as those who had smoked ≥400 cigarettes during their lifetime but did not smoke at the time of the survey, and on-going smokers were defined as smokers who had smoked ≥400 cigarettes during their lifetime and still smoked cigarettes at the time of the survey. Among the ever-smokers, including both quitters and on-going smokers, information was collected on age of smoking initiation, number of cigarettes smoked per day, and smoking duration by posing the following questions: “At what age did you start smoking?”, “How many years have you been smoking?”, and “On average, how many cigarettes do you smoke per day?” The respondents who reported that they no longer smoked were asked: “At what age did you stop smoking?” or “How long ago did you stop smoking?” To estimate lifetime tobacco exposure (i.e. smoking intensity), pack-years were calculated as the average number of cigarettes smoked per day multiplied by the number of years smoked divided by 20 (unit of one pack). Pack-years were classified into four categories: ≤10, 11–20, 21–30 and >30.

Secondhand smoke exposure was determined by asking: “How many times do you indirectly inhale smoke from other people at home?” and “How many times do you indirectly inhale smoke from other people at your workplace?” The respondents who reported that they do not indirectly inhale smoke either at home or at work were classified as the unexposed group for secondhand smoke, and those who said they indirectly inhaled smoke at least once a week or more, regardless of their location, were defined as the exposed group.

### Other putative predictors

Through a literature review, we selected the potential predictors in the smoking-related cycle from initiation to cessation as follows: age, obesity, marriage, educational attainment, occupation, drinking status and disease morbidity such as cardiovascular diseases (e.g. stroke, myocardial infarction and hypertension), diabetes mellitus, respiratory diseases (e.g. chronic bronchitis and asthma) and cancer [15, 17, 19–22]. All these putative predictors were evaluated in the present study.

Age was categorised into four birth cohorts, 1935–39, 1940–49, 1950–59 and 1960–, which corresponded to the age groups of 65–69, 55–68, 45–58 and 40–48 years, respectively. The obese group was defined by a BMI ≥25 kg/m^2^, and those with a BMI <25 kg/m^2^ were defined as the normal group. Marital status was dichotomised into married or single and the married group included those who were cohabiting. Educational attainment was divided into three categories in the order of increasing level of education: middle school or below, high-school graduation and university degree or above. Current occupation was divided into three groups: manual (skilled agricultural, forestry and fishery workers; craft and related trades workers; plant, machine operators and assemblers; elementary occupations), non-manual (legislators, senior officials and managers, professionals and related workers, clerical support workers, service workers and sale workers) and unemployed.

Alcohol drinking history was determined by the following question: “Are you unable to consume alcohol or refuse to do so (for religious reasons, etc.)?” The respondents who have never drunk alcohol were determined as never drinkers. Drinkers, on the other hand, were defined as respondents who have ever drunk alcohol and responded either ‘yes’ (current drinkers) or ‘no’ (former drinkers) to the question, “Do you still drink?”

Past disease history was ascertained based on the responses to the separate questions: “Have you ever been diagnosed with a disease by a doctor in a hospital?” and “Are you currently undergoing any treatment for the disease?” Past disease history on stroke, myocardial infarction, hypertension, diabetes mellitus, respiratory disease and cancer was investigated separately. The subjects who reported having being diagnosed with one of these diseases were classified as the disease history positive group and those who had never been diagnosed with any of these diseases were classified as the disease history negative group.

### Statistical analysis

To compare the basic characteristics across categories of smoking status, a chi-square test and one-way analysis of variance (ANOVA) were conducted. To investigate which factors were significantly associated with smoking initiation and/or smoking cessation, two hierarchical multivariate logistic regression models were used to estimate odds ratios (ORs) and 95% confidence intervals (CIs), accounting for demographic characteristics and past disease history (Model 1) and demographic characteristics, past disease history and smoking-related history (Model 2). To diminish the chance effect by quitters who returned to smoking, all the analyses of smoking cessation were conducted in the following phases: First, all quitters were included in the statistical models, regardless of their abstinence period; Second, quitters who continued to be non-smokers for at least two years or more were defined as successful quitters and were analysed separately.

The associations between the selected predictors and the likelihood of reaching smoking cessation were assessed with Cox proportional hazards regression models. To identify which of the aforementioned predictors constituted the most important determinants of a successful smoking cessation, a stepwise selection method was applied with a 0.05 significance level for entry and elimination. Among the chosen determinants, estimates of interaction effect were calculated on a multiplicative scale. Furthermore, Kaplan–Meier curves were constructed to illustrate the distribution of time from age at smoking initiation to age at smoking cessation. Smoking cessation was defined as the main outcome measure, and on-going smokers were treated as censored data. Statistical differences between the curves were estimated using the log rank test. All statistical analyses were performed using SAS software version 9.3 (SAS Institute, Cary, North Carolina).

## Results

The subjects’ characteristics, including demographic characteristics, disease history and smoking-related history, are summarised in Table 1. The following seemed to be associated with being a non-smoker: having a high educational qualification, having a non-manual job, being a never drinker and not being exposed to secondhand smoke (*p*<0.001). Quitters showed a significantly higher proportion of the following: obesity (43.5%), marriage (95.2%), past alcohol consumption (12.4%) and history of diseases, such as stroke (2.4%), myocardial infarction (4.7%), hypertension (24.7%), diabetes mellitus (10.1%), respiratory disease (3.2%) and cancer (2.7%, Table 1).

**Table 1:** Basic characteristics of the study population.

		Non-smokers (N = 8,417)	Quitters (N = 7,886)	On-going smokers (N = 8,187)	*P*
***Demographic characteristics***					
Age	Years, mean ± SD	54.7±8.3	54.9±7.9	51.9±8.0	<0.001
Birth cohort	1935–1939	570 (6.8)	486 (6.2)	267 (3.3)	<0.001
	1940–1949	3,055 (36.3)	2,927 (37.1)	2,036 (24.9)	
	1950–1959	2,904 (34.5)	3,055 (38.7)	3,271 (39.9)	
	1960-	1,888 (22.4)	1,418 (18.0)	2,613 (31.9)	
Obesity ^a^	Normal	5,061 (60.1)	4,452 (56.5)	4,995 (61.0)	<0.001
	Obese	3,345 (39.8)	3,430 (43.5)	3,186 (38.9)	
	Unknown	11 (0.1)	4 (0.0)	6 (0.1)	
Marital status	Single	358 (4.3)	327 (4.1)	579 (7.1)	<0.001
	Married ^b^	7,967 (94.6)	7,507 (95.2)	7,537 (92.1)	
	Unknown	92 (1.1)	52 (0.7)	71 (0.8)	
Education	≤ Middle school	2,179 (25.9)	2,057 (26.1)	2,191 (26.8)	<0.001
	High school graduate	3,005 (35.7)	3,228 (40.9)	3,579 (43.7)	
	≥ University degree	3,029 (36.0)	2,499 (31.7)	2,264 (27.7)	
	Unknown	204 (2.4)	102 (1.3)	153 (1.8)	
Occupation	Manual	3,656 (43.4)	3,528 (44.7)	4,386 (53.6)	<0.001
	Non-manual	2,736 (32.5)	2,344 (29.7)	2,324 (28.4)	
	Unemployed	1,619 (19.2)	1,673 (21.2)	1,187 (14.5)	
	Not defined	406 (4.8)	341 (4.3)	290 (3.5)	
Alcohol drinking	Current drinkers	5,025 (59.7)	5,695 (72.2)	6,614 (80.8)	<0.001
	Ex-drinkers	475 (5.6)	975 (12.4)	370 (4.5)	
	Never drinkers	2,902 (34.5)	1,208 (15.3)	1,196 (14.6)	
	Unknown	15 (0.2)	8 (0.1)	7 (0.1)	
***Disease history***					
Stroke		155 (1.8)	100 (2.4)	108 (1.3)	<0.001
Myocardial infarction		274 (3.3)	370 (4.7)	204 (2.5)	<0.001
Hypertension		1,920 (22.8)	1,951 (24.7)	1,381 (16.9)	<0.001
Diabetes mellitus		721 (8.6)	795 (10.1)	675 (8.2)	<0.001
Respiratory disease ^c^		213 (2.5)	253 (3.2)	182 (2.2)	0.002
Cancer		129 (1.5)	214 (2.7)	81 (1.0)	<0.001
***Smoking-related history***					
Secondhand smoke	Unexposed	6,285 (74.7)	4,839 (61.4)	4,629 (56.5)	<0.001
	Exposed at home/work	2,048 (24.3)	3,000 (38.0)	3,504 (42.8)	
	Unknown	84 (1.0)	47 (0.6)	54 (0.7)	
Smoking initiation age	Years, mean ± SD	- ^d^	21.3±4.1	21.9±5.0	<0.001
Smoking duration	Years, mean ± SD	- ^d^	23.7±10.3	29.1±9.1	<0.001
Smoking dose/day	Cigarettes, mean ± SD	- ^d^	18.1±9.9	17.8±8.5	0.107
Lifetime tobacco exposure	mean ± SD	- ^d^	22.2±16.7	26.2±15.2	<0.001

a. Body mass index < 25 kg/m^2^ (normal) *vs*. body mass index ≥ 25 kg/m^2^ (obese)

b. Married people including cohabitants

c. Ever diagnosed with chronic bronchitis and/or asthma

d. Not applicable for non-smokers

To identify putative determinants of smoking initiation, ever-smokers were compared with the never smokers (Table 2). After accounting for all the selected predictors, the greatest likelihood of smoking onset was significantly associated with the youngest cohort (OR = 1.46, 95% CI 1.27–1.67), ex-drinkers (OR = 1.17, 95% CI 1.05–1.31) and morbidity related to several diseases (OR = 1.26, 95% CI 1.08–1.47 for myocardial infarction; OR = 1.16, 95% CI 1.05–1.28 for diabetes; and OR = 1.42, 95% CI 1.14–1.78 for cancer). Secondhand smoke exposure showed 1.91-times increased odds for smoking initiation (95% CI 1.79–2.03). On the contrary, subjects who were married (OR = 0.83, 95% CI 0.73–0.94), had a higher socioeconomic position (OR = 0.84, 95% CI 0.77–0.91 for a university or higher degree; OR = 0.86, 95% CI 0.80–0.93 for a non-manual occupation), and were never drinkers (OR = 0.36, 95% CI 0.34–0.38) showed decreased odds for smoking initiation (Table 2).

**Table 2:** Odds ratios (95% confidence intervals) comparing those who initiate smoking with never smokers: non-smokers *vs*. ever smokers.

		Non-smokers (N = 8,417)	Ever smokers (N = 16,073)	OR (95% CI) ^a^	OR (95% CI) ^b^
***Demographic characteristics***					
Birth cohort	1935–1939	570 (6.8)	753 (4.7)	1 (reference)	1 (reference)
	1940–1949	3,055 (36.3)	4,963 (30.9)	1.23 (1.09–1.38)	1.17 (1.03–1.32)
	1950–1959	2,904 (34.5)	6,326 (39.4)	1.65 (1.47–1.85)	1.51 (1.33–1.72)
	1960-	1,888 (22.4)	4,031 (25.1)	1.62 (1.43–1.83)	1.46 (1.27–1.67)
Obesity ^c^	Normal	5,061 (60.1)	9,447 (58.8)	1 (reference)	1 (reference)
	Obese	3,345 (39.8)	6,616 (41.2)	1.06 (1.00–1.12)	1.04 (0.99–1.10)
Marital status	Single	358 (4.3)	906 (5.6)	1 (reference)	1 (reference)
	Married ^d^	7,967 (94.6)	15,044 (93.6)	0.75 (0.66–0.85)	0.83 (0.73–0.94)
Education	≤ Middle school	2,179 (25.9)	4,248 (26.4)	1 (reference)	1 (reference)
	High school graduate	3,005 (35.7)	6,807 (42.4)	1.16 (1.09–1.24)	1.10 (1.02–1.18)
	≥ University degree	3,029 (36.0)	4,763 (29.6)	0.81 (0.75–0.86)	0.84 (0.77–0.91)
Occupation	Manual	3,656 (43.4)	7,914 (49.2)	1 (reference)	1 (reference)
	Non-manual	2,736 (32.5)	4,668 (29.0)	0.79 (0.74–0.84)	0.86 (0.80–0.93)
	Unemployed	1,619 (19.2)	2,860 (17.8)	0.82 (0.76–0.88)	1.10 (1.01–1.19)
Alcohol drinking	Current drinkers	2,902 (34.5)	2,404 (15.0)	1 (reference)	1 (reference)
	Ex-drinkers	475 (5.6)	1,345 (8.4)	1.16 (1.04–1.29)	1.17 (1.05–1.31)
	Never drinkers	5,025 (59.7)	12,309 (76.6)	0.34 (0.32–0.36)	0.36 (0.34–0.38)
***Disease history***					
Stroke		155 (1.8)	297 (1.9)	1.00 (0.83–1.22)	1.10 (0.89–1.35)
Myocardial infarction		274 (3.3)	574 (3.6)	1.10 (0.95–1.27)	1.26 (1.08–1.47)
Hypertension		1,920 (22.8)	3,332 (20.7)	0.88 (0.83–0.94)	0.86 (0.80–0.92)
Diabetes mellitus		721 (8.6)	1,470 (9.2)	1.07 (0.98–1.18)	1.16 (1.05–1.28)
Respiratory disease ^e^		213 (2.5)	435 (2.7)	1.07 (0.91–1.27)	1.14 (0.96–1.35)
Cancer		129 (1.5)	295 (1.8)	1.20 (0.98–1.48)	1.42 (1.14–1.78)
***Smoking-related history***					
Secondhand smoke	Unexposed	6,285 (74.7)	9,468 (58.9)	1 (reference)	1 (reference)
	Exposed at home/work	2,048 (24.3)	6,504 (40.5)	2.11 (1.99–2.24)	1.91 (1.79–2.03)

Unknown values were wholly included in the statistical models but were not presented in the table.

a. Adjusted for birth cohort, obese status, marital status, smoking status, education, occupational classification, alcohol drinking, and history of diseases

b. Adjusted for birth cohort, obese status, marital status, smoking status, education, occupational classification, alcohol drinking, history of diseases, and secondhand smoke exposure

c. Body mass index < 25 kg/m^2^ (normal) *vs*. body mass index ≥ 25 kg/m^2^ (obese)

d. Married peoples including cohabitants

e. Ever diagnosed with chronic bronchitis and/or asthma

The factors associated with successful smoking cessation are shown in Table 3. The odds of successfully quitting smoking increased with being married (OR = 1.73, 95% CI 1.47–2.04), achieving higher education level (OR = 1.56, 95% CI 1.40–1.74), having a non-manual occupation (OR = 1.24, 95% CI 1.13–1.36), drinking cessation (OR = 2.53, 95% CI 2.21–2.90) and disease morbidity (OR = 1.36, 95% CI 1.12–1.65 for myocardial infarction; OR = 1.32, 95% CI 1.21–1.44 for hypertension; and OR = 1.88, 95% CI 1.41–2.49 for cancer). In contrast, younger age and secondhand smoke exposure were associated with a lower likelihood of smoking cessation (OR = 0.25, 95% CI 0.21–0.30 and OR = 0.88, 95% CI 0.82–0.95). Moreover, a continual decrease in the odds of successfully quitting smoking was observed according to increased age at smoking initiation, smoking duration, smoking dose per day and lifetime tobacco exposure (*p* trend <0.001, Table 3).

**Table 3:** Odds ratios (95% confidence intervals) comparing successful quitters with on-going smokers.

		Abstinence for > 2 years *vs*. current smokers				All quitters *vs*. current smokers			
		Quitters (N = 6,130)	On-going (N = 8,187)	OR (95% CI) ^a^	OR (95% CI) ^b^	Quitters (N = 7,886)	On-going (N = 8,187)	OR (95% CI) ^a^	OR (95% CI) ^b^
***Demographic characteristics***									
Birth cohort	1935–1939	387 (6.3)	267 (3.3)	1 (reference)	1 (reference)	486 (6.2)	267 (3.3)	1 (reference)	1 (reference)
	1940–1949	2,410 (39.3)	2,036 (24.9)	0.84 (0.71–1.00)	0.81 (0.68–0.97)	2,927 (37.1)	2,036 (24.9)	0.82 (0.69–0.96)	0.79 (0.67–0.93)
	1950–1959	2,330 (38.0)	3,271 (39.9)	0.54 (0.45–0.64)	0.49 (0.41–0.59)	3,055 (38.7)	3,271 (39.9)	0.56 (0.47–0.66)	0.52 (0.44–0.61)
	1960-	1,003 (16.4)	2,613 (31.9)	0.28 (0.24–0.34)	0.25 (0.21–0.30)	1,418 (18.0)	2,613 (31.9)	0.32 (0.27–0.39)	0.29 (0.24–0.35)
Obesity ^c^	Normal	3,508 (57.2)	4,995 (61.0)	1 (reference)	1 (reference)	4,452 (56.5)	4,995 (61.0)	1 (reference)	1 (reference)
	Obese	2,619 (42.7)	3,186 (38.9)	1.16 (1.08–1.24)	1.16 (1.08–1.25)	3,430 (43.5)	3,186 (38.9)	1.20 (1.13–1.29)	1.21 (1.13–1.29)
Marital status	Single	237 (3.9)	579 (7.1)	1 (reference)	1 (reference)	327 (4.1)	579 (7.1)	1 (reference)	1 (reference)
	Married ^d^	5,859 (95.6)	7,537 (92.1)	1.71 (1.45–2.01)	1.73 (1.47–2.04)	7,507 (95.2)	7,537 (92.1)	1.61 (1.39–1.87)	1.63 (1.41–1.89)
Education	≤ Middle school	1,582 (25.8)	2,191 (26.8)	1 (reference)	1 (reference)	2.057 (26.1)	2,191 (26.8)	1 (reference)	1 (reference)
	High school graduate	2,481 (40.5)	3,579 (43.7)	1.17 (1.07–1.28)	1.20 (1.10–1.31)	3,228 (40.9)	3,579 (43.7)	1.14 (1.27–1.55)	1.17 (1.08–1.27)
	≥ University degree	1,989 (32.5)	2,264 (27.7)	1.49 (1.34–1.66)	1.56 (1.40–1.74)	2,499 (31.7)	2,264 (27.7)	1.40 (1.27–1.55)	1.46 (1.32–1.61)
Occupation	Manual	2,697 (44.0)	4,386 (53.6)	1 (reference)	1 (reference)	3,528 (44.7)	4,386 (53.6)	1 (reference)	1 (reference)
	Non-manual	1,833 (29.9)	2,324 (28.4)	1.23 (1.13–1.35)	1.24 (1.13–1.36)	2,344 (29.7)	2,324 (28.4)	1.22 (1.12–1.33)	1.23 (1.13–1.34)
	Unemployed	1,357 (22.1)	1,187 (14.5)	1.16 (1.04–1.28)	1.16 (1.04–1.29)	1,673 (21.2)	1,187 (14.5)	1.15 (1.04–1.27)	1.15 (1.05–1.28)
Alcohol drinking	Current drinkers	4,420 (72.1)	6,614 (80.8)	1 (reference)	1 (reference)	5,695 (72.2)	6,614 (80.8)	1 (reference)	1 (reference)
	Ex-drinkers	753 (12.3)	370 (4.5)	2.58 (2.25–2.95)	2.53 (2.21–2.90)	975 (12.4)	370 (4.5)	2.62 (2.31–2.99)	2.59 (2.28–2.95)
	Never drinkers	950 (15.5)	1,196 (14.6)	1.10 (1.00–1.21)	1.10 (1.00–1.22)	1,208 (15.3)	1,196 (14.6)	1.10 (1.00–1.20)	1.10 (1.00–1.20)
***Disease history***									
Stroke		150 (2.5)	108 (1.3)	1.20 (0.92–1.56)	1.18 (0.91–1.54)	189 (2.4)	108 (1.3)	1.20 (0.94–1.55)	1.19 (0.92–1.53)
Myocardial infarction		283 (4.6)	204 (2.5)	1.39 (1.14–1.68)	1.36 (1.12–1.65)	370 (4.7)	204 (2.5)	1.43 (1.19–1.71)	1.40 (1.17–1.69)
Hypertension		1,563 (25.5)	1,381 (16.9)	1.31 (1.20–1.44)	1.32 (1.21–1.44)	1,951 (24.7)	1,381 (16.9)	1.28 (1.18–1.40)	1.29 (1.18–1.40)
Diabetes mellitus		627 (10.2)	675 (8.2)	0.86 (0.76–0.98)	0.86 (0.76–0.98)	795 (10.1)	675 (8.2)	0.88 (0.79–0.99)	0.88 (0.79–0.99)
Respiratory disease ^e^		204 (3.3)	182 (2.2)	1.22 (0.99–1.51)	1.21 (0.98–1.49)	253 (3.2)	182 (2.2)	1.21 (0.99–1.48)	1.19 (0.98–1.46)
Cancer		162 (2.6)	81 (1.0)	1.95 (1.47–2.59)	1.88 (1.41–2.49)	214 (2.7)	81 (1.0)	2.02 (1.54–2.64)	1.95 (1.49–2.55)
***Smoking-related history***									
Secondhand smoke	Unexposed	3,843 (62.7)	4,629 (56.5)	1 (reference)	1 (reference)	4,839 (61.4)	4,629 (56.5)	1 (reference)	1 (reference)
	Exposed	2,251 (36.7)	3,504 (42.8)	0.88 (0.82–0.94)	0.88 (0.82–0.95)	3,000 (38.0)	3,504 (42.8)	0.92 (0.86–0.98)	0.92 (0.85–0.98)
Smoking initiation age	< 20 years old	1,740 (28.4)	1,986 (24.3)	1 (reference)	1 (reference)	2,227 (28.2)	1,986 (24.3)	1 (reference)	1 (reference)
	20–24	3,321 (54.2)	4,497 (54.9)	0.76 (0.70–0.82)	0.82 (0.74–0.91)	4,269 (54.1)	4,497 (54.9)	0.77 (0.71–0.83)	0.82 (0.75–0.91)
	25–29	766 (12.5)	1,052 (12.9)	0.64 (0.57–0.73)	0.79 (0.65–0.95)	975 (12.4)	1,052 (12.8)	0.67 (0.60–0.74)	0.79 (0.66–0.93)
	≥ 30 years old	303 (4.9)	652 (7.9)	0.37 (0.32–0.43)	0.55 (0.40–0.75)	415 (5.3)	652 (8.0)	0.41 (0.35–0.47)	0.56 (0.42–0.75)
				*P*_trend_ < 0.001	*P*_trend_ < 0.001			*P*_trend_ < 0.001	*P*_trend_ < 0.001
Smoking duration	≤ 10 years	994 (16.2)	218 (2.7)	1 (reference)	1 (reference)	1,036 (13.1)	218 (2.7)	1 (reference)	1 (reference)
	11–20	1,947 (31.8)	1,176 (14.4)	0.38 (0.32–0.45)	0.24 (0.20–0.29)	2,220 (28.2)	1,176 (14.4)	0.42 (0.35–0.49)	0.29 (0.24–0.34)
	21–30	2,086 (34.0)	3,637 (44.4)	0.12 (0.10–0.14)	0.06 (0.05–0.08)	2,818 (35.7)	3,637 (44.4)	0.16 (0.14–0.19)	0.09 (0.08–0.11)
	> 30	1,103 (18.0)	3,156 (38.5)	0.05 (0.04–0.06)	0.02 (0.02–0.03)	1,812 (23.0)	3,156 (38.5)	0.09 (0.07–0.10)	0.05 (0.04–0.05)
				*P*_trend_ < 0.001	*P*_trend_ < 0.001			*P*_trend_ < 0.001	*P*_trend_ < 0.001
Smoking dose per day	≤ 10 cigarettes	2,062 (33.6)	2,205 (26.9)	1 (reference)	1 (reference)	2,546 (32.3)	2,205 (26.9)	1 (reference)	1 (reference)
	11–20	3,120 (50.9)	4,834 (59.1)	0.73 (0.68–0.79)	0.47 (0.41–0.53)	4,137 (52.5)	4,834 (59.1)	0.78 (0.73–0.84)	0.52 (0.46–0.58)
	> 20	948 (15.5)	1,148 (14.0)	0.97 (0.87–1.09)	0.34 (0.26–0.44)	1,203 (15.2)	1.148 (14.0)	0.98 (0.88–1.08)	0.37 (0.29–0.47)
				*P*_trend_ = 0.006	*P*_trend_ < 0.001			*P*_trend_ = 0.021	*P*_trend_ < 0.001
Lifetime tobacco exposure	≤ 10 pack-years	1,828 (29.8)	1,040 (12.7)	1 (reference)	1 (reference)	2,041 (25.9)	1,040 (12.7)	1 (reference)	1 (reference)
	11–20	1,817 (29.6)	2,083 (25.4)	0.49 (0.44–0.54)	0.24 (0.22–0.27)	2,277 (28.9)	2,083 (25.4)	0.55 (0.50–0.61)	0.32 (0.28–0.35)
	21–30	1,282 (20.9)	2,501 (30.6)	0.28 (0.25–0.31)	0.08 (0.07–0.09)	1,787 (22.7)	2,501 (30.6)	0.35 (0.32–0.39)	0.13 (0.12–0.15)
	> 30	1,203 (19.6)	2,563 (31.3)	0.22 (0.20–0.25)	0.03 (0.02–0.03)	1,781 (22.6)	2,563 (31.3)	0.30 (0.27–0.34)	0.06 (0.05–0.07)
				*P*_trend_ < 0.001	*P*_trend_ < 0.001			*P*_trend_ < 0.001	*P*_trend_ < 0.001

Unknown values were wholly included in the statistical models but were not presented in the table.

a. Adjusted for birth cohort, obese status, marital status, smoking status, education, occupational classification, alcohol drinking, and history of diseases

b. Adjusted for birth cohort, obese status, marital status, smoking status, education, occupational classification, alcohol drinking, history of diseases, secondhand smoke exposure, smoking initiation age, and smoking dose

c. Body mass index < 25 kg/m^2^ (normal) *vs*. body mass index ≥ 25 kg/m^2^ (obese)

d. Married people including cohabitants

e. Ever diagnosed with chronic bronchitis and/or asthma

Through a stepwise selection process, lifetime tobacco exposure, educational attainment, alcohol drinking status and birth cohort were identified as the major determinants of a successful smoking cessation. Among the predictors, lifetime tobacco exposure had the greatest association (inverse) with the likelihood of successful quitting (β = -0.6141 and β = -0.5282, Table 4). Furthermore, a significant interaction of birth cohort with educational attainment, secondhand smoke, and lifetime tobacco exposure was exhibited (*p* = 0.007, *p* = 0.012, and *p* < 0.001, respectively, S1 Table).

**Table 4:** Predictors increasing the likelihood of achieving smoking cessation by Cox proportional hazard models ^a^.

	*β*	*Standard error*	*P*
**Abstinence for > 2 years *vs*. current smokers**			
Lifetime tobacco exposure (ref. ≤ 10 pack-years)	-0.6141	0.0127	<0.001
Alcohol drinking status (ref. Ex-drinkers)	-0.2139	0.0189	<0.001
Birth cohort (ref. 1935–1939)	0.1162	0.0184	<0.001
Educational attainment (ref. Lowest level—middle school or below)	0.0687	0.0125	<0.001
**All quitters *vs*. current smokers**			
Lifetime tobacco exposure (ref. ≤ 10 pack-years)	-0.5282	0.0112	<0.001
Birth cohort (ref. 1935–1939)	0.3987	0.0174	<0.001
Alcohol drinking (ref. Ex-drinkers)	-0.1947	0.0166	<0.001
Educational attainment (ref. Lowest level—middle school or below)	0.0654	0.0109	<0.001
Smoking initiation age (ref. < 20 years old)	0.0375	0.0031	<0.001

a. Smoking cessation was defined as the main outcome measure, and on-going smokers were treated as censored data in fully adjusted models

**Fig 1:**
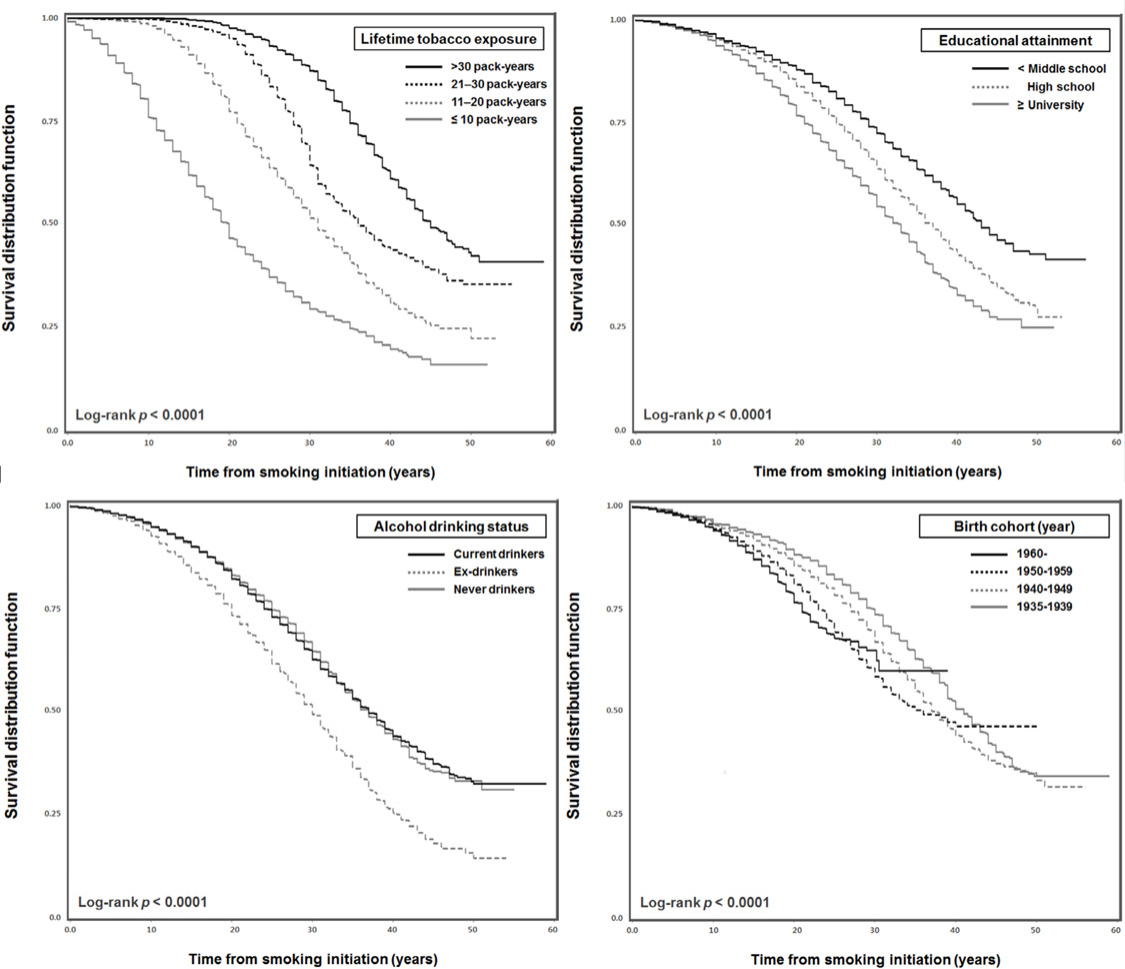
Kaplan–Meier estimates for successful smoking cessation according to the selected predictors. In the restricted analysis with quitters who continued to be non-smokers for at least two years or more and on-going smokers.

Fig 1 presents the estimates for the time from smoking initiation to cessation according to the selected major determinants. Under a restricted analysis, which included only the quitters who remained non-smokers for at least two years or more, at 30 years after the initiation of smoking, smokers who were exposed to a greater amount of lifetime tobacco smoke had a substantially lower estimated rate of quitting than those who had less exposure (*p* <0.0001). It was estimated that only 27.7% of smokers who did not graduate from middle school would quit smoking 30 years later compared with 42.8% of smokers who attained a university degree or higher (*p* <0.0001). Ex-drinkers were more likely to quit smoking within 30 years, with 48.6% smokers converting to non-smokers compared with smokers who had never drunk in their lifetime or still drank (33.2% and 35.2%). Interestingly, when the results of the analysis of the absolute smoking period during the entire lifetime were considered, it was estimated that the younger birth cohort significantly tended to be non-smokers 30 years later, compared with the older age groups (37.7%, 39.7%, 30.5% and 26.4%, respectively, in the order of the younger birth cohort, Fig 1).

**Fig 2:**
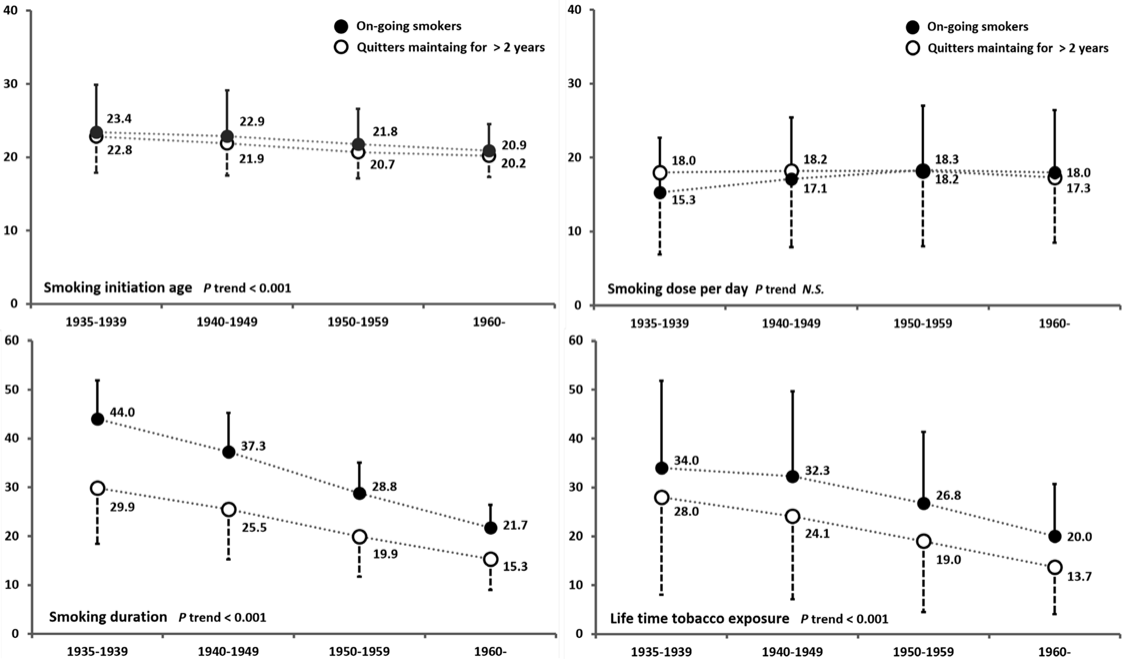
Smoking-related history according to birth cohorts. Black circles represent on-going smokers and white circles represent quitters who continued to be non-smokers for at least two years or more.

Differences in smoking-related history according to each birth cohort and smoking status were also evaluated. The older birth cohorts appeared to have initiated smoking at a later age but had smoked for a longer duration. The mean smoking dose per day did not distinguish the older and younger generations (among quitters) or showed a slightly lower dose (among on-going smokers). However, the absolute intensity of exposure to cigarette smoking was higher among the older birth cohorts (*p* trend <0.001, Fig 2).

## Discussion

Smoking behaviour is the result of multifactorial influences and varies across the life course.

Thus, diverse factors (i.e. social, economic, environmental, behavioural and physiological) may affect smoking trajectories. In Korea—a developed non-Western country—lifetime tobacco exposure, educational attainment, alcohol drinking status and birth cohort appeared to be the potent determinants of a successful smoking cessation: those factors have also been consistently reported as significant predictors of smoking cessation in Western countries. Moreover, the likelihood of being a successful quitter was changed by marriage, occupational classification, disease morbidity and secondhand smoke exposure.

Smokers who have been exposed to larger amounts of lifetime tobacco smoke appeared to have a substantially lower likelihood of changing their smoking status. In the present study, a significant association was observed between cessation and increased smoking duration, daily smoking dose and lifetime tobacco exposure. Moreover, smokers who were exposed to a larger amount of lifetime tobacco smoke needed much longer time to become non-smokers. These results are consistent with those of others, which found that daily cigarette consumption per day, a longer duration of smoking history and greater inhalation were more likely to postpone smoking cessation [23], while smoking fewer cigarettes per day strongly predicted successful quitting [19]. Researches have also suggested that the success or failure of smoking cessation can be mediated by the intensity of nicotine exposure, which is considered to be the most potent contributor to cigarette smoking [24, 25]. Nicotine is generally assumed to be a considerable barrier to cessation, and studies have indicated that the large majority of on-going smokers frequently fail to overcome nicotine withdrawal symptoms despite their intention to quit [2, 12, 16, 26–28]. All evidence supports that understanding the individual’s history of lifetime tobacco smoke can be the starting point to improve smoking cessation outcomes, regardless of whether smokers live in Western or non-Western countries. Such knowledge will aid in coupling smokers with tailor-made intervention programs, which are more likely to assist cessation of smoking.

Secondhand smoke exposure seems to be strongly associated with the initiation, maintenance and cessation of smoking. Our findings indicated that there is an increased likelihood of smoking initiation and less likelihood of smoking cessation among those with secondhand smoke exposure. This implies that individuals who are exposed to secondhand smoke may live under the smoker-friendly circumstances and may show more generous attitudes toward smoking behaviour as a result. They may have increased opportunities for tobacco use and may be easily exposed to smoking triggers during the period of smoking cessation. In the same vein, abundant evidence has demonstrated that the smoking behaviour of members from individuals’ social network (i.e., parents, siblings, peers, and neighbourhood) can be a major determinant of smoking uptake [29–32] and that smokers in social settings with many other smokers find it difficult to stop smoking [15, 17]. Given that non-smokers may start cigarette smoking simply due to social interactions with smokers, in establishing smoking cessation and prevention activities, particular attention must be paid to socially disadvantaged groups that are exposed to secondhand smoke. Such groups represent the most vulnerable population at the bottom of the smoking chain.

Socioeconomic disadvantages, particularly with regard to educational attainment, appear to be a crucial predictor of smoking behaviours. Studies have reported that 1) lower educational achievement is significantly associated with smoking initiation [33, 34], 2) highly educated smokers are more likely to attempt quitting smoking [35], and 3) highly educated smokers show higher cessation rates [36, 37]: such phenomenon is consistently apparent in the Western populations. Our results from a developed non-Western population support the evidence that educational attainment is a predominant factor mediating an individual’s smoking behaviour. Given that educational attainment can reflect overall socioeconomic status at the individual level and health-related knowledge [38], more attention must be paid to individuals with low socioeconomic status and enhanced interventions are also needed to help this group to quit smoking and to refrain from smoking initiation [22]. In addition, although we could not assess the direct impact of social influences on smoking cessation due to lacking information, socially disadvantaged individuals appear to be commonly exposed to circumstances that induce smoking continuation, such as various stressors related to financial strain, lack of social support to quit, secondhand smoke exposure at home and socialising with smokers [34, 39–41]; and this impact may be more dominant for younger age groups. There’s still a need for studies focused on a social gradient different from each birth cohort. Moreover, smoke-free programs targeting socially disadvantaged groups should adopt a holistic approach to smoking-related cycles across the life course.

The co-occurrence of cigarette smoking and alcohol drinking is frequently observed, and they are assumed to be intimately correlated. Research has shown that smokers are much more likely to be alcohol drinkers and that drinkers are much more likely to be smokers [42]. Comorbidity between nicotine dependence and alcohol dependence has been also documented [43]. Although there are several inconsistencies in the literature, studies have typically found that alcohol consumption is associated with unsuccessful smoking cessation, with studies showing that 1) smoking cessation is less likely to be sustained in the presence of alcohol consumption [11, 44] and that 2) less frequent alcohol consumption is significantly associated with a higher likelihood of quitting smoking [45]. Our findings also support the negative association between alcohol drinking and successful smoking cessation. Furthermore, in the present study, ex-drinkers exhibited higher odds of being quitters than never drinkers. This can be explained by the possibility of hidden triggers, such as newly developed diseases and/or dramatic incidents, leading to increased concerns on health and the tendency toward the prohibition of smoking and drinking altogether. Indeed, a significantly higher proportion of disease morbidity was observed among quitters, in addition to increased likelihoods of smoking cessation and drinking abstinence (S2 and S3 Tables). Further studies should be conducted to interpret the underlying dynamics of the transition from smoking to non-smoking in smokers with different physical conditions and drinking patterns.

Almost uniformly, the odds of successfully quitting smoking have been reported to increase with age [15, 19, 45]. Studies have generally explained this finding in terms of functional impairment and/or medication initiation among older smokers that result in them quitting cigarette smoking [19, 23]. In particular, traumatic events, along with physical restriction and/or psychological shock, appeared to strongly motivate older smokers to give up smoking [13, 23]. Our findings regarding the ORs are consistent with those of others. However, when we considered the analysis of the absolute smoking period during the entire lifetime, the younger age groups do not appear to remain smoking for as long as the older age groups. Furthermore, there exhibits a significant interaction of birth cohorts with smoking-related behaviours. This indicates that 1) birth cohort can influence on personal norms and attitudes about smoking; and 2) the different perception on smoking by each age group may induce various differences in smoking initiation, continuation, and termination and finally, determine cumulative lifetime exposure of smoking. In the present study, the older smokers were more likely to be exposed to a higher intensity of lifetime tobacco smoking than the younger smokers; even more, the older quitters had already smoked for a relatively longer time compared with the younger smokers who still smoked cigarettes every day. Whereas, it is possibly assumed that the smoking career of young smokers would be finished earlier than that of the old generation even if they had started smoking at a much earlier age. To understand the complexity in smoking trajectories across generations, an integrated approach embracing the different birth cohort effect connected with the social norms about smoking and behavioural outcomes is warranted.

The following limitations should be noted. First, the cross-sectional analysis precludes us from establishing a causal relationship or speculating the chronological changes in smoking trajectories. Due to the limitation of cross-sectional design, we could not explore the actual dynamics of smoking behaviour. Second, the subjects’ smoking history, as well as other factors, were ascertained based on self-reporting methods; thus, the present study may not be free of information bias. Third, due to a lack of detailed information on smoking cessation, such as the total number of attempts to quit and use of nicotine replacement therapy, we were unable to explore the dynamics of smoking relapse. Finally, the current study cannot draw conclusions about the success or failure of quitting smoking based on the depth of inhalation or the nicotine content per cigarettes. Furthermore, we were unable to adopt biomonitoring information on nicotine addiction due to the restricted data access. Thus, the findings should be interpreted with caution. Further follow-up studies embracing biological responses and longitudinal changes should be warranted to confirm the association in the holistic process from smoking initiation to complete cessation. Despite these limitations, the present study is based on one of the largest cohorts in Korea and explores the crucial factors involved in smoking initiation and termination among the middle- and older-aged male population. Moreover, the inclusion of various factors potentially associated with smoking-related behaviours in this large-scale cross-sectional analysis enabled us to identify the most important determinants in the success of smoking cessation. Our findings can provide a context for successful smoking cessation programs in developed non-Western (i.e. Asian) settings as well as in Korea.

## Conclusion

Lifetime tobacco exposure, educational attainment, alcohol drinking status and birth cohort could be potent determinants in the success of smoking cessation, regardless of whether smokers live in Western or non-Western countries. Moreover, diverse factors, such as marriage, occupational classification, disease morbidity and secondhand smoke exposure, may play a role in the initiation and/or termination of smoking. Considering most of the factors selected in the present study have been consistently replicated as significant predictors of smoking cessation in Western countries, it is suggested that public interventions adopted by Western nations—i.e., regulatory strategies encouraged by the WHO Framework Convention on Tobacco Control (FCTC)—may also help us to reinforce discouraging the initiation of, reducing, and quitting cigarette smoking and eventually promote a smoke-free nation.

## Supporting Information

S1 Table Likelihoods of reaching smoking cessation by Cox proportional hazard models: stratified analysis by birth cohorts. (DOCX)

S2 Table Past disease history according to smoking and drinking status. (DOCX)

S3 Table Association between past disease history and combined status of cigarette smoking and alcohol drinking. (DOCX)
