# Supplementary material for: What Are the Major Determinants in the Success of Smoking Cessation: Results from the Health Examinees Study
Source: PLoS One. 2015 Dec 3;10(12):e0143303. doi: 10.1371/journal.pone.0143303 (PMC4669113; doi:10.1371/journal.pone.0143303)
Supplement: S1 Table — (DOCX) [file pone.0143303.s001.docx]

**S1 Table. Likelihoods of reaching smoking cessation by Cox proportional hazard models ^a^: stratified analysis by birth cohorts**

|  |  | **Birth cohort strata** | | | |  |
| --- | --- | --- | --- | --- | --- | --- |
|  | **Total subjects**  **HR (95% CI)** | **1935-1939**  **HR (95% CI)** | **1940-1949**  **HR (95% CI)** | **1950-1959**  **HR (95% CI)** | **1960-**  **HR (95% CI)** | ***P***  **_interaction_** |
| ***Demographic characteristics*** |  |  |  |  |  |  |
| **Birth cohort** |  |  |  |  |  |  |
| 1935-1939 | 1 (reference) |  |  |  |  |  |
| 1940-1949 | 1.14 (1.03-1.28) |  |  |  |  |  |
| 1950-1959 | 1.42 (1.26-1.60) |  |  |  |  |  |
| 1960- | 1.45 (1.27-1.66) |  |  |  |  |  |
| **Obesity ^b^** |  |  |  |  |  |  |
| Normal | 1 (reference) | 1 (reference) | 1 (reference) | 1 (reference) | 1 (reference) | 0.080 |
| Obese | 1.14 (1.08-1.20) | 1.14 (0.92-1.42) | 1.15 (1.06-1.25) | 1.14 (1.05-1.24) | 1.12 (0.99-1.28) |  |
| **Marital status** |  |  |  |  |  |  |
| Single | 1 (reference) | 1 (reference) | 1 (reference) | 1 (reference) | 1 (reference) | 0.211 |
| Married ^c^ | 1.42 (1.24-1.62) | 1.33 (0.78-2.27) | 1.16 (0.94-1.43) | 1.69 (1.33-2.13) | 1.46 (1.10-1.93) |  |
| **Education** |  |  |  |  |  |  |
| Middle school or below | 1 (reference) | 1 (reference) | 1 (reference) | 1 (reference) | 1 (reference) | 0.007 |
| High school graduate | 1.09 (1.02-1.16) | 1.41 (1.09-1.81) | 1.10 (1.00-1.22) | 0.95 (0.85-1.05) | 0.99 (0.78-1.26) |  |
| University degree or higher | 1.22 (1.13-1.32) | 1.66 (1.25-2.20) | 1.33 (1.19-1.49) | 0.98 (0.86-1.11) | 1.03 (0.80-1.32) |  |
| **Occupation** |  |  |  |  |  |  |
| Manual | 1 (reference) | 1 (reference) | 1 (reference) | 1 (reference) | 1 (reference) | 0.808 |
| Non-manual | 1.13 (1.06-1.21) | 0.87 (0.56-1.36) | 1.26 (1.12-1.43) | 1.21 (1.10-1.34) | 0.93 (0.80-1.07) |  |
| Unemployed | 1.03 (0.96-1.11) | 1.08 (0.85-1.39) | 1.08 (0.98-1.19) | 0.94 (0.80-1.10) | 0.77 (0.52-1.15) |  |
| **Alcohol drinking** |  |  |  |  |  |  |
| Current drinkers | 1 (reference) | 1 (reference) | 1 (reference) | 1 (reference) | 1 (reference) | 0.417 |
| Ex-drinkers | 1.66 (1.53-1.80) | 1.38 (1.02-1.86) | 1.70 (1.52-1.91) | 1.59 (1.38-1.82) | 1.96 (1.51-2.54) |  |
| Never drinkers | 1.12 (1.04-1.20) | 0.96 (0.73-1.27) | 1.07 (0.96-1.19) | 1.18 (1.05-1.33) | 1.22 (1.00-1.48) |  |
| ***Disease history*** |  |  |  |  |  |  |
| **Stroke** | 1.10 (0.93-1.30) | 0.61 (0.35-1.08) | 1.16 (0.93-1.45) | 1.19 (0.88-1.61) | 1.01 (0.90-1.37) | 0.823 |
| **Myocardial infarction** | 1.20 (1.06-1.35) | 1.30 (0.87-1.93) | 1.14 (0.96-1.34) | 1.25 (1.01-1.56) | 1.42 (0.86-2.36) | 0.822 |
| **Hypertension** | 1.13 (1.07-1.21) | 1.33 (1.07-1.65) | 1.05 (0.96-1.15) | 1.27 (1.15-1.41) | 1.11 (0.90-1.37) | 0.986 |
| **Diabetes mellitus** | 0.98 (0.90-1.06) | 1.27 (0.96-1.66) | 0.94 (0.83-1.05) | 0.89 (0.76-1.05) | 1.28 (0.91-1.79) | 0.893 |
| **Respiratory disease ^e^** | 1.17 (1.02-1.35) | 1.38 (0.92-2.07) | 1.12 (0.91-1.37) | 1.25 (0.97-1.60) | 0.92 (0.56-1.52) | 0.931 |
| **Cancer** | 1.16 (0.99-1.37) | 0.74 (0.42-1.30) | 1.06 (0.85-1.32) | 1.58 (1.19-2.10) | 1.96 (1.10-3.49) | 0.427 |
| ***Smoking-related history*** |  |  |  |  |  |  |
| **Secondhand smoke** |  |  |  |  |  |  |
| Non-exposed | 1 (reference) | 1 (reference) | 1 (reference) | 1 (reference) | 1 (reference) | 0.012 |
| Exposed at home/work | 0.96 (0.91-1.00) | 0.77 (0.61-0.98) | 0.98 (0.90-1.07) | 0.96 (0.88-1.04) | 0.91 (0.80-1.03) |  |
| **Lifetime tobacco exposure** |  |  |  |  |  |  |
| ≤ 10 pack-years | 1 (reference) | 1 (reference) | 1 (reference) | 1 (reference) | 1 (reference) | <0.001 |
| 11–20 | 0.46 (0.43-0.49) | 0.48 (0.35-0.66) | 0.45 (0.40-0.51) | 0.48 (0.43-0.53) | 0.39 (0.34-0.45) |  |
| 21–30 | 0.27 (0.25-0.29) | 0.32 (0.23-0.44) | 0.36 (0.32-0.41) | 0.25 (0.22-0.28) | 0.13 (0.10-0.15) |  |
| > 30 | 0.16 (0.15-0.18) | 0.23 (0.17-0.31) | 0.20 (0.18-0.22) | 0.13 (0.11-0.15) | 0.14 (0.10-0.19) |  |

Unknown values were wholly included in the statistical models but were not presented in the table

1. Smoking cessation was defined as the main outcome measure, and current smokers were treated as censored data in fully adjusted models which included only the quitters who remained non-smokers for at least two years or more
2. Body mass index < 25 kg/m^2^ (normal) *vs.* body mass index ≥ 25 kg/m^2^ (obese)
3. Married people including cohabitants
4. Ever diagnosed with chronic bronchitis and/or asthma
