# Supplementary material for: What Are the Major Determinants in the Success of Smoking Cessation: Results from the Health Examinees Study
Source: PLoS One. 2015 Dec 3;10(12):e0143303. doi: 10.1371/journal.pone.0143303 (PMC4669113; doi:10.1371/journal.pone.0143303)
Supplement: S2 Table — (DOCX) [file pone.0143303.s002.docx]

**S2 Table. Past disease history according to smoking and drinking status**

|  | **Smoking status** | | | |  | **Drinking status ^a^** | | | |
| --- | --- | --- | --- | --- | --- | --- | --- | --- | --- |
|  | **Non-smokers**  **(N=8,417)** | **Quitters**  **(N=7,886)** | **On-going smokers**  **(N=8,187)** | ***P*** |  | **Non-drinkers**  **(N=5,306)** | **Quitters**  **(N=1,820)** | **On-going drinkers**  **(N=17,334)** | ***P*** |
| **Stroke** | 155 (1.8) | 100 (2.4) | 108 (1.3) | <0.001 |  | 112 (2.1) | 106 (5.8) | 234 (1.4) | <0.001 |
| **Myocardial infarction** | 274 (3.3) | 370 (4.7) | 204 (2.5) | <0.001 |  | 233 (4.4) | 123 (6.8) | 491 (2.8) | <0.001 |
| **Hypertension** | 1,920 (22.8) | 1,951 (24.7) | 1,381 (16.9) | <0.001 |  | 1,011 (19.1) | 481 (26.4) | 3,758 (21.7) | <0.001 |
| **Diabetes mellitus** | 721 (8.6) | 795 (10.1) | 675 (8.2) | <0.001 |  | 512 (9.7) | 272 (15.0) | 1,405 (8.1) | <0.001 |
| **Respiratory disease ^b^** | 213 (2.5) | 253 (3.2) | 182 (2.2) | 0.002 |  | 175 (3.3) | 77 (4.2) | 396 (2.3) | <0.001 |
| **Cancer** | 129 (1.5) | 214 (2.7) | 81 (1.0) | <0.001 |  | 121 (2.3) | 114 (6.3) | 189 (1.1) | <0.001 |

1. Among a total of 24,490 study population, 30 subjects with unavailable information on drinking status were excluded in this combined analysis
2. Ever diagnosed with chronic bronchitis and/or asthma
