# Supplementary material for: What Are the Major Determinants in the Success of Smoking Cessation: Results from the Health Examinees Study
Source: PLoS One. 2015 Dec 3;10(12):e0143303. doi: 10.1371/journal.pone.0143303 (PMC4669113; doi:10.1371/journal.pone.0143303)
Supplement: S3 Table — (DOCX) [file pone.0143303.s003.docx]

**S3 Table. Association between past disease history and combined status of cigarette smoking and alcohol drinking**

|  | **Combined status with cigarette smoking and alcohol drinking (N=24,460) ^a^** | | | | | | | | | | |  |
| --- | --- | --- | --- | --- | --- | --- | --- | --- | --- | --- | --- | --- |
|  | **Non-smokers** | | |  | **Quitters** | | |  | **On-going smokers** | | |  |
|  | **Non-drinkers**  **(N=2,902)** | **Quitters**  **(N=475)** | **On-going**  **(N=5,025)** |  | **Non-drinkers**  **(N=1,208)** | **Quitters**  **(N=975)** | **On-going**  **(N=5,695)** |  | **Non-drinkers**  **(N=1,196)** | **Quitters**  **(N=370)** | **On-going**  **(N=6,614)** | ***P*** |
| **Stroke** | 64 (2.2) | 23 (4.8) | 68 (1.4) |  | 35 (2.9) | 67 (6.9) | 87 (1.5) |  | 13 (1.1) | 16 (4.3) | 79 (1.2) | <0.001 |
| **Myocardial infarction** | 110 (3.8) | 23 (4.8) | 140 (2.8) |  | 79 (6.5) | 79 (8.1) | 212 (3.7) |  | 44 (3.7) | 21 (5.7) | 139 (2.1) | <0.001 |
| **Hypertension** | 613 (21.1) | 135 (28.4) | 1,171 (23.3) |  | 250 (20.7) | 268 (27.5) | 1,432 (25.1) |  | 148 (12.4) | 78 (21.1) | 1,155 (17.5) | <0.001 |
| **Diabetes mellitus** | 263 (9.1) | 62 (13.1) | 396 (7.9) |  | 132 (10.9) | 143 (14.7) | 519 (9.1) |  | 117 (9.8) | 67 (18.1) | 490 (7.4) | <0.001 |
| **Respiratory disease ^b^** | 91 (3.1) | 15 (3.2) | 107 (2.1) |  | 53 (4.4) | 39 (4.0) | 161 (2.8) |  | 31 (2.6) | 23 (6.2) | 128 (1.9) | <0.001 |
| **Cancer** | 53 (1.8) | 27 (5.7) | 49 (1.0) |  | 49 (4.1) | 73 (7.5) | 92 (1.6) |  | 19 (1.6) | 14 (3.8) | 48 (0.7) | <0.001 |

1. Among a total of 24,490 study population, 30 subjects with unavailable information on drinking status were excluded in this combined analysis
2. Ever diagnosed with chronic bronchitis and/or asthma
